# Umi-pipeline-nf: a modular and scalable workflow for UMI-tagged nanopore amplicon analysis with real-time sequencing integration and GPU-acceleration

**DOI:** 10.1093/bioinformatics/btag160

**Published:** 2026-04-01

**Authors:** Stephan Amstler, Lukas Forer, Lara Escherich, Sebastian Schönherr, Stefan Coassin

**Affiliations:** Institute of Genetic Epidemiology, Medical University of Innsbruck, Innsbruck 6020, Austria; Institute of Genetic Epidemiology, Medical University of Innsbruck, Innsbruck 6020, Austria; Institute of Genetic Epidemiology, Medical University of Innsbruck, Innsbruck 6020, Austria; Institute of Genetic Epidemiology, Medical University of Innsbruck, Innsbruck 6020, Austria; Institute of Genetic Epidemiology, Medical University of Innsbruck, Innsbruck 6020, Austria

## Abstract

**Motivation:**

Unique molecular identifiers (UMIs) enable efficient error correction in amplicon sequencing but UMI-aware analysis workflows for long-read sequencing and particularly for nanopore data are still sparse. Existing approaches lack portability, real-time sequencing support, GPU acceleration, and efficient use of resources.

**Results:**

We present umi-pipeline-nf, a portable, fully containerized, modular and scalable workflow to create single-molecule consensus sequences from UMI-tagged long-read nanopore amplicon data. Umi-pipeline-nf supports flexible UMI-designs and is built in Nextflow DSL2 for seamless deployment across computing platforms and a high degree of parallelization, allowing analysis of several targets at once. It scales linearly from single samples to large cohorts, outperforming existing tools in efficiency and flexibility. Additionally, we integrated real-time read processing, robust UMI clustering, and GPU-accelerated consensus polishing. Umi-pipeline-nf supports two different polishing strategies [reference sequence-based and partial order alignment (POA)-based]. Implementation of GPU-accelerated, reference sequence-based polishing results in up to 100-fold runtime improvements and reduced usage of computational resources, compared to other UMI analysis pipelines and POA-based polishing.

**Availability and implementation:**

The umi-pipeline-nf analysis pipeline and test data are available at https://github.com/genepi/umi-pipeline-nf, and a frozen snapshot is available at DOI: 10.5281/zenodo.18607956. Scripts and configuration files for the analyses in the present manuscript can be found at https://github.com/AmstlerStephan/umi-pipeline-nf_Paper.

## 1 Introduction

The single-molecule error rates of long-read sequencing (LRS) are still relatively high compared to short-read sequencing (SRS) (Wang *et al.* 2021). This limits its utility to characterize mutational patterns when assembly-based polishing is not possible, such as somatic mutations, complex, homologous genome regions, and detection of low-frequency variants (Logsdon *et al.* 2020, Wagner *et al.* 2022, Olson *et al.* 2023). In contrast, SRS lacks haplotype (i.e. phase) resolution beyond the read length of 500 bp (Wagner *et al.* 2022, Olson *et al.* 2023).

Unique molecular identifiers (UMIs) offer a robust error-correction strategy in SRS and have recently been established for LRS (Zurek *et al.* 2020, Karst *et al.* 2021, Amstler *et al.* 2024, Deng *et al.* 2024). UMIs are short, random nucleotide sequences that allow tagging of molecules with a unique molecular barcode. After amplification and sequencing, reads derived from the original molecule can be identified and clustered by their tagging UMIs (Amstler *et al.* 2024). Consensus-based polishing allows removal of PCR and sequencing errors, achieving single-molecule accuracy exceeding Q40 (<0.01% error rate) (Karst *et al.* 2021, Amstler *et al.* 2024).

By combining high single-molecule accuracy with multi-kilobases reads, UMI-based LRS is particularly useful in resolving paralogous sequence variants and VNTR units (Amstler *et al.* 2024, Deng *et al.* 2024), accurate classification in complex metagenomic mixtures (Karst *et al.* 2021, Deng *et al.* 2024), detection and phasing of low-level somatic mutations (Zurek *et al.* 2020, Kwon and Shin 2024, Zahm *et al.* 2025, Zhu *et al.* 2025), and monitoring of intra-host viral evolution patterns (Westfall *et al.* 2024, Zahm *et al.* 2025). Further applications extend into sensitive pathogen surveillance (Karst *et al.* 2021), detection and phasing of mitochondrial heteroplasmies (Payne *et al.* 2013), T-cell receptor profiling (Shen *et al.* 2023), and HLA genotyping (Cornaby *et al.* 2022).

Existing LRS UMI analysis pipelines are often proof-of-principle implementations, memory-intensive, inflexible to different UMI designs and lack support for scalable execution (Karst *et al.* 2021, Zahm *et al.* 2025). Nanopore sequencing provides real-time sequencing data of each single molecule (Burgess 2021, Kovaka *et al.* 2021, Payne *et al.* 2021, Hook and Timp 2023, Munro *et al.* 2025, Patel *et al.* 2025, van der Toorn *et al.* 2025), which allows live UMI clustering potentially improving resource and flow cell capacity management in targeted sequencing experiments (Deamer *et al.* 2016, Wang *et al.* 2021). Until now, no UMI-aware workflow uses the potential of real-time data monitoring.

Here we present umi-pipeline-nf, a modular, containerized, and reproducible workflow built in Nextflow DSL2 (Di Tommaso *et al.* 2017, Strozzi *et al.* 2019). It supports analysis for different, flexible UMI-designs, robust two-step UMI clustering, parallel processing of several targets, linear scaling independent of sample size, and two complementary polishing strategies using Medaka (https://github.com/nanoporetech/medaka) with optional GPU-acceleration.

## 2 Results

**Figure 1:**
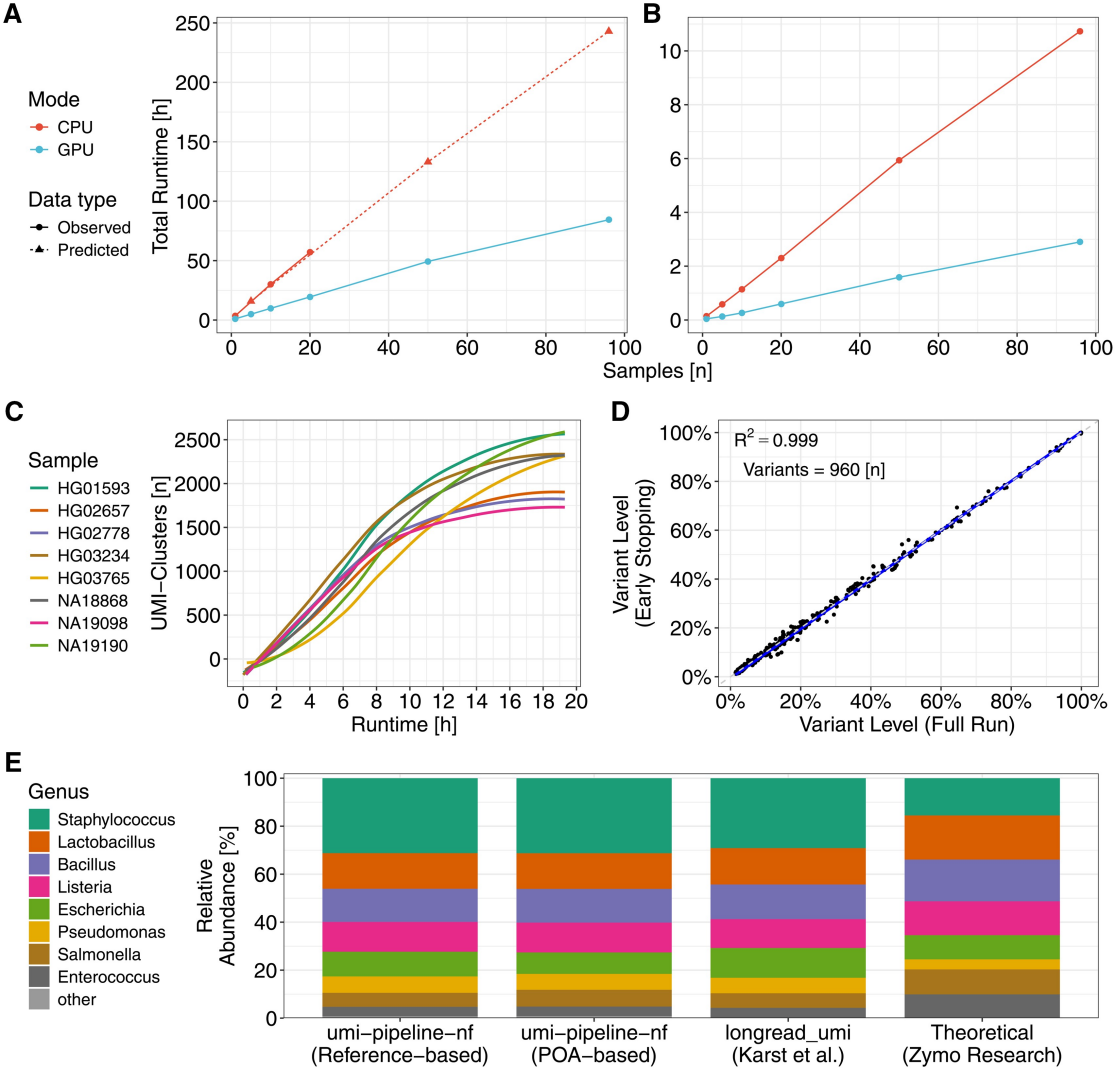
umi-pipeline-nf: Scalability, UMI-cluster stability during live monitoring, accuracy of early-stopping of a sequencing run, and benchmarking with longread_umi (Karst *et al*. 2021) with Zymo mock-community agreement. (A and B) Scalability of total runtime with increasing sample count for POA-based (A) and reference-based (B) polishing strategies. Points and lines encode mode (CPU/GPU) and data type (observed/extrapolated); *y*-axis shows total runtime of umi-pipeline-nf in hours, *x*-axis shows samples (*n*). All four strategies scale linearly with increasing sample size. (C) Number of UMI clusters during real-time monitoring of an UMI-ONT-Seq experiment of the *LPA* KIV-2 locus (Amstler *et al*. 2024) for eight samples (colored lines). After an initial delay, the number of clusters increases steadily over time, plateauing after 16 h of sequencing (LOESS-smoothed trend, individual data points shown in Fig. 6, available as supplementary data at *Bioinformatics* online). (D) Variant-level agreement between early-stopping (threshold of 500 cluster per sample) and the full *LPA* KIV-2 UMI-ONT-Seq run. Scatter plot with the identity line (dashed) and linear fit (blue) shows near-perfect concordance (*R*^2^ = 0.999; *n* = 960 variants). (E) Zymo mock-community species composition. Stacked bars compare umi-pipeline-nf (GPU reference- and POA-based), longread_umi, and the theoretical mixture levels from Zymo (see Table 7, available as supplementary data at *Bioinformatics* online). While both polishing strategies of umi-pipeline-nf correlate closely with the results of longread_umi (POA-based *R*^2^ = 0.971 and reference-based *R*^2^ = 0.985; Fig. 8, available as supplementary data at *Bioinformatics* online), all three methods deviate from the theoretical mixture levels (longread_umi *R*^2^ = 0.528, umi-pipeline-nf POA-based *R*^2^ = 0.476 and umi-pipeline-nf reference-based *r* = 0.463), which was driven primarily by two groups (Table 7, available as supplementary data at *Bioinformatics* online).

Umi-pipeline-nf provides a fast, scalable, and accurate framework for analyzing UMI-tagged nanopore amplicon data at single-molecule resolution. It performs pre-processing, UMI-aware clustering with quality control, consensus polishing [partial order alignment (POA)-based using Medaka (Lee *et al.* 2002) or reference-based, Supplementary notes, available as supplementary data at *Bioinformatics* online] and optional variant calling. The pipeline is highly configurable, supporting flexible UMI-designs and specification of anchor sequences (Supplementary notes: UMI-design considerations, available as supplementary data at *Bioinformatics* online), and UMI clusters can be tracked in real-time during sequencing (Fig. 1, available as supplementary data at *Bioinformatics* online). Further features include GPU-accelerated polishing and support for sample multiplexing. Full documentation and example datasets are available on GitHub (https://github.com/genepi/umi-pipeline-nf).

### 2.1 Portability and flexibility of umi-pipeline-nf

Implemented in Nextflow and fully containerized, umi-pipeline-nf runs reproducibly across local machines, HPC clusters, and cloud environments. It supports CPU-only and GPU infrastructures, with GPU acceleration providing substantial performance benefits (Figs 2 and 3, available as supplementary data at *Bioinformatics* online). Its modular design and comprehensive unit testing with nf-test (Forer and Schönherr 2025) and GitHub workflows allow easy maintenance and ensure robust updates.

### 2.2 POA-based polishing vs reference-based polishing

We compared POA-based and reference-based polishing in terms of runtime and memory consumption in both GPU and CPU modes using nanopore-sequenced UMI amplicon data for the *LPA* KIV-2 VNTR (Amstler *et al.* 2024) from a sample of the 1000 Genomes Project (HG00653). All experiments were performed using the UMI-design from Amstler *et al.* (Amstler *et al.* 2024) (Supplementary methods, available as supplementary data at *Bioinformatics* online).

By default, Medaka performs POA-based sequence alignment for each UMI cluster prior polishing. This is independent of a reference sequence but computationally demanding. In contrast, for use cases where a reference sequence is available, using the reference-based strategy reduces polishing time and RAM consumption by up to ≈30-fold compared to POA-based polishing (Tables 1 and 2, available as supplementary data at *Bioinformatics* online and Fig. 2A–C, available as supplementary data at *Bioinformatics* online; testing workstation specifications given in the Supplementary methods, available as supplementary data at *Bioinformatics* online). GPU acceleration provided ≈4-fold speed-up (Table 3, available as supplementary data at *Bioinformatics* online). We observed virtually identical variant calling results for all four strategies (Table 4, available as supplementary data at *Bioinformatics* online).

We also assessed how the performance of the different strategies scales with increasing number of clusters per polishing input file (i.e. the FASTA/FASTQ input files containing grouped UMI clusters for polishing) and the maximum number of reads per UMI cluster. CPU POA-based polishing execution times increased non-linearly with file size (Fig. 3A and B, available as supplementary data at *Bioinformatics* online), while CPU reference-based and GPU-accelerated POA-based polishing runtimes scaled linearly (Fig. 3C–E, available as supplementary data at *Bioinformatics* online). GPU acceleration of reference-based polishing led to near-constant polishing times (Fig. 3F, available as supplementary data at *Bioinformatics* online). With increasing cluster number per polishing input file, the overall runtime decreased for both reference-based polishing configurations (Fig. 4A and B, available as supplementary data at *Bioinformatics* online), stayed constant for GPU POA-based polishing (Fig. 4C, available as supplementary data at *Bioinformatics* online) and increased for CPU POA-based polishing (Fig. 4D, available as supplementary data at *Bioinformatics* online).

In summary, support for GPU acceleration provides expected performance increases, while reference-based polishing substantially reduces the execution time and RAM consumption without compromising variant calling in our test case. POA-based polishing is reference-independent for applications where no suitable reference sequence is available.

### 2.3 Scalability

Next, we evaluated how umi-pipeline-nf scales across increasing sample numbers by running both POA-based and reference-based polishing strategies on nanopore-sequenced UMI amplicon data for the *LPA* KIV-2 VNTR (Amstler *et al.* 2024) of 1 to 96 samples (Supplementary methods, available as supplementary data at *Bioinformatics* online). Runtime increased linearly with the number of samples and clusters for both strategies (Fig. 1A and B; Table 5, available as supplementary data at *Bioinformatics* online) and reference-based polishing showed an about 70-fold decrease in normalized runtime compared to the POA-based polishing (Fig. 5B, available as supplementary data at *Bioinformatics* online). Umi-pipeline-nf demonstrates robust, linear scaling and applicability to large sample sets.

### 2.4 Real-time nanopore sequencing integration

Implementation of umi-pipeline-nf in Nextflow DSL2 allows monitoring of an ongoing nanopore sequencing run and therefore real-time analysis of UMI-clusters. New reads are automatically subjected to pre-processing, UMI detection, and clustering. Real-time updates of cluster statistics allow users to stop sequencing and trigger cluster polishing once an adequate number of UMI clusters is achieved. We include a simple demo of the live monitoring in the umi-pipeline-nf GitHub repository (https://github.com/genepi/umi-pipeline-nf/tree/main/live_demo).

To validate cluster stability over time, we monitored a full UMI-ONT-Seq run for the *LPA* KIV-2 VNTR (Amstler *et al.* 2024) consisting of 8 samples of the 1000 Genomes Project (CPU reference-based polishing). After an initial delay, cluster number increased continuously and plateauing started at 1200–2500 cluster per sample after about 16 h (Fig. 1C). After 19.3 h, the full sequencing run was stopped and resulted in 8.9 Gb (5.4 M reads) and 1754–2607 clusters per sample (mean ± SD: 2233 ± 334; Table 6, available as supplementary data at *Bioinformatics* online; Fig. 6A, available as supplementary data at *Bioinformatics* online).

To validate early stopping of a sequencing run based on the number of clusters we performed a second sequencing run with the same flowcell and library. The early stopping sequencing run was stopped after 10 h (3 Gb sequencing data, 1.8 M reads) at minimum 500 clusters per sample (mean ± SD: 896 ± 207 clusters, Fig. 6B, available as supplementary data at *Bioinformatics* online and table 6, available as supplementary data at *Bioinformatics* online). Using the full run as a reference, recall, precision, specificity, and F1-score for the found variants were all 100%, with the variant levels being highly correlated (*R*^2^ = 0.999 ± 0.002; mean ± SD; Fig. 1D and Fig. 7, available as supplementary data at *Bioinformatics* online).

### 2.5 Benchmarking

In contrast to existing UMI pipelines, umi-pipeline-nf is modular, portable, containerized, and supports two different polishing strategies optimized for different use cases and flexible definition of the UMI design. Unfortunately, two out of three existing UMI pipelines could not be executed on our own data (Amstler *et al.* 2024) due to RAM constraints or inflexible, hard-coded UMI designs. Therefore, we used umi-pipeline-nf to reanalyze each of the datasets used in the evaluation of the other pipelines by the respective authors [*longread_umi* (Karst *et al.* 2021), *ConSeqUMI* (https://github.com/JGEnglishLab/ConSeqUMI) (Zahm *et al.* 2025), and *pipeline-umi-*amplicon (https://github.com/nanoporetech/pipeline-umi-amplicon)], applying the default settings of each pipeline.

We reanalyzed the Zymo mock community data from Karst *et al.* [*longread_umi* (Karst *et al.* 2021)] with both umi-pipeline-nf polishing strategies. We used the original UMI design and the universal amplification primer as anchor sequence to locate the start of the UMI sequences (Supplementary methods, available as supplementary data at *Bioinformatics* online). Both strategies were highly correlated with the longread_umi results (*R*^2^ = 0.971 for POA-based and *R*^2^ = 0.985 for reference-based polishing, Fig. 8, available as supplementary data at *Bioinformatics* online). Reference-based polishing yielded 5% more classifiable reads compared to POA-based polishing (≈47 000 vs. ≈45 000, Table 7, available as supplementary data at *Bioinformatics* online). The additional reads were evenly added across all species, without adding extra noise (Table 7, available as supplementary data at *Bioinformatics* online). The GPU-accelerated reference-based analysis with umi-pipeline-nf required 55 CPU hours, compared to ≈500 CPU hours for longread_umi. Noteworthy, while we reproduce the results of longread_umi, all three methods, however, deviate from the theoretical abundance, as already discussed in Karst *et al.* (2021) (Fig. 1E, Table 7, available as supplementary data at *Bioinformatics* online).

As the test dataset provided by Oxford Nanopore Technologies’ *pipeline-umi-amplicon* was too small for a meaningful evaluation, we applied it to the aforementioned *LPA* KIV-2 VNTR dataset and compared the variant calls with those from our pipeline. Non-UMI-based variant calls from the VNTR-specific variant caller *vntr-calling-nf* (Di Maio *et al.* 2024) were used as reference method (Supplementary methods, available as supplementary data at *Bioinformatics* online). All three pipelines showed highly consistent variant calls for high-frequency variants, but pipeline-umi-amplicon additionally reported >700 low-level false positives in the analyzed 5.1 kb amplicon (Fig. 9, available as supplementary data at *Bioinformatics* online). Variant levels detected by all three pipelines and both umi-pipeline-nf settings correlated strongly (*R*^2^ = 0.878 to *R*^2^ = 1, Fig. 10A-F, available as supplementary data at *Bioinformatics* online), but low-level variants detected by pipeline-umi-amplicon showed a large variance (Fig. 10B, D, and F, available as supplementary data at *Bioinformatics* online), as reported in (Amstler *et al.* 2024).

Finally, we benchmarked umi-pipeline-nf against *ConSeqUMI* using SARS-CoV-2 data from Zahm *et al.*, which evaluated intra-host viral evolution in four patients (Zahm *et al.* 2025). They used the same UMI design as in Karst *et al.* (2021), but different amplification primer, which we used as context sequence (Supplementary methods, available as supplementary data at *Bioinformatics* online). Viral lineage assignment was performed using Nextclade (Hadfield *et al.* 2018). In line with ConSeqUMI, umi-pipeline-nf confirmed that all four samples contained only Delta variants, including a mixed 21A/J and 21I population in one patient (Table 8, available as supplementary data at *Bioinformatics* online). Both pipelines detected a nonsynonymous substitution (I834V) usually found in Omicron lineages (Table 9, available as supplementary data at *Bioinformatics* online) (Gangavarapu *et al.* 2023). Both identified A222V and V367L only in 21I subclades, but umi-pipeline-nf did not confirm them as a single haplotype nor as occurring uniformly across all 21I subclades (Table 9, available as supplementary data at *Bioinformatics* online). Also, a previously undescribed back mutation at residue I882 detected by ConSeqUMI was not found by umi-pipeline-nf (Table 9, available as supplementary data at *Bioinformatics* online) (Gangavarapu *et al.* 2023, Zahm *et al.* 2025).

## 3 Discussion

Umi-pipeline-nf is a modular Nextflow workflow for processing UMI-tagged nanopore amplicon data that supports GPU-accelerated consensus polishing and robust two-step UMI clustering. Umi-pipeline-nf matches the accuracy of existing pipelines while providing substantial improvements in flexibility for different UMI designs, speed, resource efficiency, scalability, and reproducibility.

Real-time monitoring of the UMI clusters during the sequencing run presents a unique and practical advance over offline workflows. After careful validation of the cluster size threshold required for the specific research question, this feature may help reduce sequencing costs and optimize flow cell usage by allowing the runs to be stopped once sufficient coverage is achieved.

POA-based polishing is reference-independent but computationally expensive. In contrast, reference-based polishing, especially with GPU acceleration, enables rapid, highly resource-efficient polishing of even large sample sets, when a sufficiently similar reference is available (as in most targeted sequencing applications). Conversely, POA-based polishing may be preferable for environmental samples or samples with complex mutation patterns (e.g. tumors). We observed no differences in variant calling performance on our test dataset but recommend careful validation of new targets.

Umi-pipeline-nf reproduced the results of existing pipelines, using different sets of UMI-designs and experimental setups. The pipeline provides a flexible, scalable, and reproducible framework to analyze UMI-tagged nanopore sequencing data. Importantly, UMI-based experiments need comprehensive validation with orthogonal methods, as limitations such as PCR-bias and jackpotting effects can occur (Karst *et al.* 2021, Deng *et al.* 2024, Sun *et al.* 2024). UMI-based sequencing approaches are particularly useful where high-quality (≤0.01% error-rate) single molecule sequencing at clonal resolution is necessary such as for disentangling paralogous sequence variants [e.g. between *SMN1* and *SMN2* (Vezain *et al.* 2010), *HSPA1A* and *HSPA1B* (Wadsworth *et al.* 2025) or *CR1 (**Ebbert et al.* 2019)], resolving variants in VNTR regions (Amstler *et al.* 2024), metagenomics at single base resolution [e.g. gut microbiomes (Karst *et al.* 2021)], monitoring of intra-host viral evolution [e.g. HIV (Ode *et al.* 2024), SARS-CoV2 (Zahm *et al.* 2025)], pathogen surveillance (Bejaoui *et al.* 2025) and detection of mitochondrial heteroplasmy (Fazzini *et al.* 2021). As LRS quality advances rapidly, the number of reads per UMI cluster to produce error-free consensus sequences will decrease further, making UMI experiments even more effective and opening new research opportunities across research fields.

## Supplementary Material

btag160_Supplementary_Data

## Data Availability

All sequencing data generated for the present manuscript has been uploaded to the European Nucleotide archive (ENA) Project PRJEB73509 (accessions with “first_public” time stamps 23 October 2025, 11 February 2026, and 12 February 2026). The umi-pipeline-nf pipeline and test data are available at https://github.com/genepi/umi-pipeline-nf and a frozen snapshot of the code is available at Zenodo under DOI: 10.5281/zenodo.18607956. Scripts and configuration files for the analyses in the present manuscript can be found at https://github.com/AmstlerStephan/umi-pipeline-nf_Paper.
