# Supplementary material for: Umi-pipeline-nf: a modular and scalable workflow for UMI-tagged nanopore amplicon analysis with real-time sequencing integration and GPU-acceleration
Source: Bioinformatics. 2026 Apr 1;42(4):btag160. doi: 10.1093/bioinformatics/btag160 (PMC13070649; doi:10.1093/bioinformatics/btag160)
Supplement: btag160_Supplementary_Data [file btag160_supplementary_data.pdf]

Supplementary Material for

Umi-pipeline-nf: A modular and scalable workflow for UMI-tagged nanopore amplicon analysis with real-time sequencing integration and GPU-acceleration

Stephan Amstler[a], Lukas Forer[a], Lara Escherich[a],
Sebastian Schönherr[a], Stefan Coassin[a]

[a] Institute of Genetic Epidemiology, Medical University of Innsbruck, Innsbruck, Austria

This PDF file includes

- Supplementary notes
- Supplementary methods
- Supplementary figures
- Supplementary tables
- Supplementary references

Supplementary notes

Workflow details of umi-pipeline-nf

Pre-processing and mapping

Raw nanopore sequencing reads are filtered for length and quality using catfishq[1] and optionally subsampled with seqtk[2]. A FASTA file with one or multiple reference sequences and a BED file define the target regions. Filtered reads are chunked, aligned with minimap2[3] and filtered to retain only full-length, on-target reads (default: 90% overlap with the reference). Reads are split by their reference sequence to enable parallel processing of each target (e.g. for environmental targets or amplicon mixtures).

UMI detection, clustering and quality control

UMI patterns and optional anchor sequences (i.e. the 5' part of the UMI-primer with the universal sequence for amplification of the tagged molecules[4]) are detected from both ends of each read within a configurable window (default: 200 bp). By default, 3 bp edit distance are allowed to account for sequencing errors[4]. For reads with UMIs at both ends, UMI patterns are extracted and exported in FASTA format. The sequence of the read of origin and its quality are annotated in the FASTA header of each UMI pair. The UMIs are clustered using vsearch[5], followed by graph-based refinement using NetworkX (default: edit distance 2 bp) to ensure that each cluster contains only a single UMI combination (see Supplementary Notes). High-confidence clusters above the cluster-size threshold (default: 6 reads[4]) are parsed for polishing with Medaka. Processing and filtering of the clusters are logged in a tab-separated report (.tsv).

Polishing strategies and GPU acceleration

The UMI clusters are chunked and then polished using the nanopore-specific polishing tool Medaka, with support for GPU acceleration. Umi-pipeline-nf implements two polishing strategies: The default polishing strategy of Medaka uses a reference-independent partial-order alignment (POA[6]) for polishing of each UMI cluster. The reference-based method also uses Medaka for polishing but aligns each UMI cluster to the respective reference sequence prior to polishing. This allows efficient mapping and polishing by creating a consensus over the read pileup of the alignment. While reference-based polishing supports only one round of polishing, it substantially reduces runtime and resource requirements.

Variant calling

The consensus sequences of each target of a sample can optionally be subjected to variant calling with freebayes[7], lofreq[8] or mutserve[9]. When POA-based polishing is selected, variants are called for both polishing rounds.

Principles of POA-based and reference-based UMI-cluster polishing

POA-based polishing is implemented via Medaka's smolecule workflow[10]. Reads within a UMI cluster are first aligned to each other using a Partial Order Alignment (POA) algorithm. POA is a graph-based multiple sequence alignment method in which each base forms a node in a directed acyclic graph (DAG). Alternative paths through the graph represent substitutions, insertions, or deletions. This efficiently captures variation among reads while preserving positional context. The POA alignment is then traversed to generate an initial cluster consensus, which is propagated into Medaka's neural network for base-level correction. This polishing mode retains the terminal UMI sequences within the alignment and output consensus, enabling an optional second round of polishing. Because each cluster is processed independently, POA-based polishing is highly accurate even for sequences lacking a close reference or containing extensive structural variation but is computationally more intensive in terms of time and memory.

Reference-based polishing aligns all reads in a UMI cluster directly to the corresponding target reference sequence rather than to each other. This uses minimap2 for rapid long-read mapping, followed by generation of a consensus sequence by Medaka's consensus and stitch modules. By mapping the reads to a known reference coordinate system, this approach bypasses the computational overhead of building a POA graph, which reduces RAM usage up to 5-fold and polishing time up to 30-fold. Reference-based polishing is best suited for datasets where runtime efficiency is prioritized and a reference sequence that is sufficiently similar to the sequenced target is available. Alignment against a reference needs to be carefully validated in cases of large indels, rearrangements and large heterogeneity within the target sequences.

Design considerations

The first proof-of-principle workflow for UMI analysis with nanopore sequencing was implemented as a set of bash scripts (*longread_umi*[11]), followed by a Snakemake-based UMI analysis pipeline developed specifically for nanopore sequencing by Oxford Nanopore Technologies (*pipeline-umi-amplicon*[12]). Using *pipeline-umi-amplicon* as a conceptual reference, we implemented umi-pipeline-nf in Nextflow and performed a complete redesign and functional extension. Umi-pipeline-nf is a fundamentally restructured and extensively optimized pipeline to analyse UMI-tagged nanopore data[13], incorporating new modules, GPU-accelerated polishing, two different polishing strategies, real-time sequencing integration, enhanced scalability, simple portability and improved usability.

UMI-design considerations

Although analysis of UMI-tagged nanopore sequencing data with umi-pipeline-nf produces nearly error-free consensus sequences, there are several points to be considered when designing a UMI experiment[4]. The UMI design complexity effectively limits the number of possible input molecules, as too many input molecules could cause UMI collisions and generating chimeric clusters consisting of several original input molecules[11]. Additionally, we recommend a nanopore-specific UMI design that avoids homopolymer regions and is complex enough to be uniquely occurring in the adapter sequence, as suggested by ONT[14]. Building on the UMI design suggestions from ONT we comprehensively validated this design in the complex *LPA* KIV-2 locus, adding locus specific primers to the UMIs[4]. This design was also used in all wet-lab experiments performed for the evaluation of umi-pipeline-nf. The forward UMI sequence is TTTVVVVTTVVVVTTVVVVTTVVVVTTT and the reverse sequence AAABBBBAABBBBAABBBBAABBBBAAA.

Parsing and subclustering of the UMI Clusters created by Vsearch using NetworkX

After an initial clustering of the UMI sequences by vsearch (Cluster UMIs in Supplementary figure 1), a graph-based filtering step ensures that only highly similar reads remain grouped together within a subcluster (Parse Cluster in Supplementary figure 1). Each read is represented as a node in an undirected graph, and an edge is drawn between two nodes if their pairwise edit distance (calculated using edlib) is less than or equal to a user-defined threshold (`max_edit_dist`). This creates a network in which reads are connected through chains of small sequence differences, allowing indirect links between reads that may not be identical but share high similarity via intermediate nodes. Connected components in this graph correspond to subclusters, each representing a coherent group of reads with mutual similarity. This method prevents unrelated sequences from being combined into a consensus by removing low-similarity connections that might occur in noisy or heterogenous clusters. By adapting the edit distance threshold, the stringency of this filtering can be tuned to the expected error profile of the sequencing dataset.

Supplementary methods

ONT-UMI-Seq of POA- and reference-based polishing strategies

One sample (HG00653) from the 1000 Genomes[15] (1000G) Project, included in the DNA Panel MGP00017 that was obtained from the Coriell NHGRI Sample Repository. The sample was randomly picked from this DNA panel and sequenced using our previously validated UMI-ONT-Seq[4] approach on a PromethION P2 Solo system using SQK-LSK114 chemistry. UMI-ONT-Seq amplicon data was basecalled using dorado model 400_dna_r10.4.1_e8.2_400bps_sup@v4.3.0. The bash script used to run the pipeline for all tested configurations and the according configuration files can be found at https://github.com/AmstlerStephan/umi-pipeline-nf_Paper/tree/main/benchmarking .

Specifications of the workstation used for testing

All tests were performed on a workstation running an AMD Ryzen 9 3950X AM4 16×3.5 GHz CPU with 32 cores, 64 GB DDR4 RAM and a GeForce RTX 4080 SUPER 16GB GDDR6X GPU, with an Corsair 8 TB M2.SSD hard disk.

Scalability of umi-pipeline-nf

To evaluate the scalability of umi-pipeline-nf, we benchmarked both POA-based and reference-based polishing strategies across increasing numbers of samples. For this experiment, we used a set of 96 samples drawn from the 1000 Genomes Project. Subsets of 1, 5, 10, 20, 50, 96 samples were created, with each subset representing an independent run of the complete umi-pipeline-nf workflow. The datasets were sequenced using the *LPA* KIV-2 UMI-ONT-Seq protocol[4] and basecalled with dorado model 400_dna_r10.4.1_e8.2_400bps_sup@v4.3.0. The raw reads are available in the ENA database under accession PRJEB73509. All experiments were executed using the same pipeline configuration to ensure comparability. The bash scripts and configuration files required to reproduce the benchmarking runs are available at https://github.com/AmstlerStephan/umi-pipeline-nf_Paper/tree/main/scalability. Runtime was extracted using the start time of the first process and the end time of the last process in the Nextflow trace file, and normalized runtime comparisons were made between the POA- and reference-based strategies. Due to its long execution time, CPU POA-based polishing was executed for 1, 10 and 20 samples and execution time for 5, 50 and 96 samples was extrapolated using a log-log regression accounting for a potential non-linear scaling.

Benchmarking of umi-pipeline-nf compared to longread_umi

To benchmark umi-pipeline-nf we downloaded UMI-tagged nanopore sequencing data for the ZymoBIOMICS Microbial Community DNA Standard sequenced on a MinION R10 flowcell and basecalled using guppy v3.4.4 with HAC model from the European Nucleotide repository (ENA, Accession number ERR3813594). Before running umi-pipeline-nf we chunked the single FASTQ file using seqkit (v.2.10.0) (chunk size: 300,000) [16]. We ran umi-pipeline-nf in POA-based and reference-based, GPU-mode. We used the UMI-design from the original publication (NNNYRNNNYRNNNYRNNN) and the amplification primer sequence (fwd_context: CAAGCAGAAGACGGCATACGAGAT and rev_context: GATCTCGGTGGTCGCCGTATCATT) as anchors to map the UMI patterns. The download and chunking script as well as all analysis scripts and config files can be found at https://github.com/AmstlerStephan/umi-pipeline-nf_Paper/tree/main/Karst.

Benchmarking of umi-pipeline-nf against pipeline-umi-amplicon

The same HG00653 sample from the 1000 Genomes Project[15], included in DNA Panel MGP00017 obtained from the Coriell NHGRI Sample Repository, was used for benchmarking against pipeline-umi-amplicon. UMI-ONT-Seq amplicon data generated as described above (raw reads are available in the ENA database under accession PRJEB73509) were processed with umi-pipeline-nf and pipeline-umi-amplicon. The UMI-design in this experiment was TTTVVVVTTVVVVTTVVVVTTVVVVTTT (fwd UMI) and AAABBBBAABBBBAABBBBAABBBBAAA (rev). The unambiguity of this UMI design alleviates the need for anchoring sequences. Variant calls were compared using vntr-calling-nf[17] as an independent reference. The results using 50 cluster per polishing input file and a maximum of 50 reads per UMI-cluster were used for both GPU-accelerated umi-pipeline-nf polishing strategies. The bash script used to run all tested configurations and the corresponding configuration files are available at https://github.com/AmstlerStephan/umi-pipeline-nf_Paper/tree/main/pipeline-umi-amplicon.

Benchmarking of umi-pipeline-nf compared to ConSeqUMI

To benchmark umi-pipeline-nf against the ConSeqUMI pipeline we manually downloaded their UMI-tagged and sequenced SARS-CoV-2 genomes from five patients from NCBI Gene Expression Omnibus under accession GSE288938. We ran umi-pipeline-nf in reference-based, GPU-mode on each of the samples and analyzed the resulting consensus sequences for each sample using the latest nextclade docker image (v3.15.3[18]). We used the UMI-design from the original publication (NNNYRNNNYRNNNYRNNN) and the amplification primer sequence (fwd_context: TTGCGTTCCTAGCGCCTATATTTGT and rev_context: ATCAGTCGTCATCAGCAGTCCCAC) as anchors to map the UMI patterns. The analysis

results of the ConSeqUMI pipeline were extracted from the preprint[19]. The benchmarking plots and tables were created using RStudio with R version 4.4.0 and tidyverse (v.2.0.0). Commands to run the pipeline, the configuration files and all analysis scripts can be found at https://github.com/AmstlerStephan/umi-pipeline-nf_Paper/tree/main/ConSeqUmi.

Supplementary figures

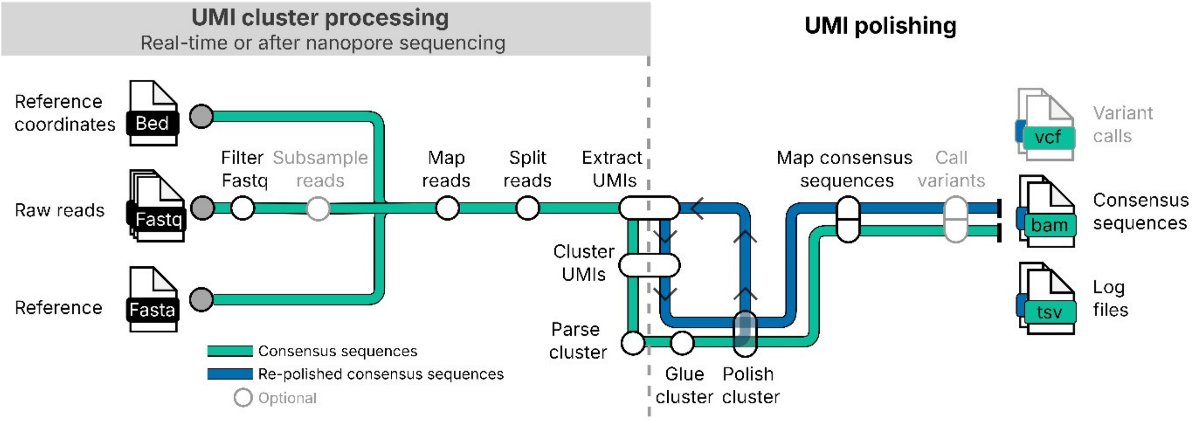

Supplementary figure 1. Metromap of umi-pipeline-nf. Input files are shown on the left side and output files on the right side. The grey box indicates steps that can be performed in real-time during nanopore sequencing.

Umi-pipeline-nf begins with quality control of UMI-tagged nanopore amplicon data (Step: *Filter Fastq*) and an optional subsampling step (*Subsample reads*). The reads are then mapped (*Map reads*) against the provided reference sequence(s) and filtered to have a user-defined overlap with the reference sequence reads (*Split reads*). Next, the UMI tags of each read are extracted (*Extract UMIs*) and clustered using vsearch[5] (*Cluster UMIs*). The UMI clusters are quality controlled using a graph-based approach to ensure integrity of the UMI pattern of each cluster and a sample-wise summary is provided (*Parse cluster*). Importantly, umi-pipeline-nf can be run live during a nanopore sequencing run up to this step, with the results being updated with every newly written FASTQ file.

After continuing, several UMI clusters are written into one file to reduce I/O-load (*Glue cluster*) and clusters are polished using Medaka either with the cluster-wise, reference-independent Partial Order Algorithm (POA)-based approach or by alignment against a reference sequence (*Polish cluster*)[10]. Cluster polishing optionally supports GPU-acceleration and POA-based polishing supports an optional second polishing round.

Finally, an optional reference- and sample-wise variant calling with three different variant callers can be performed on the UMI consensus sequences (*Map consensus sequences* and *Call variants*). Umi-pipeline-nf supports a high degree of parallelization and customization, depending on experimental design and computational resources, and is fully modularized.

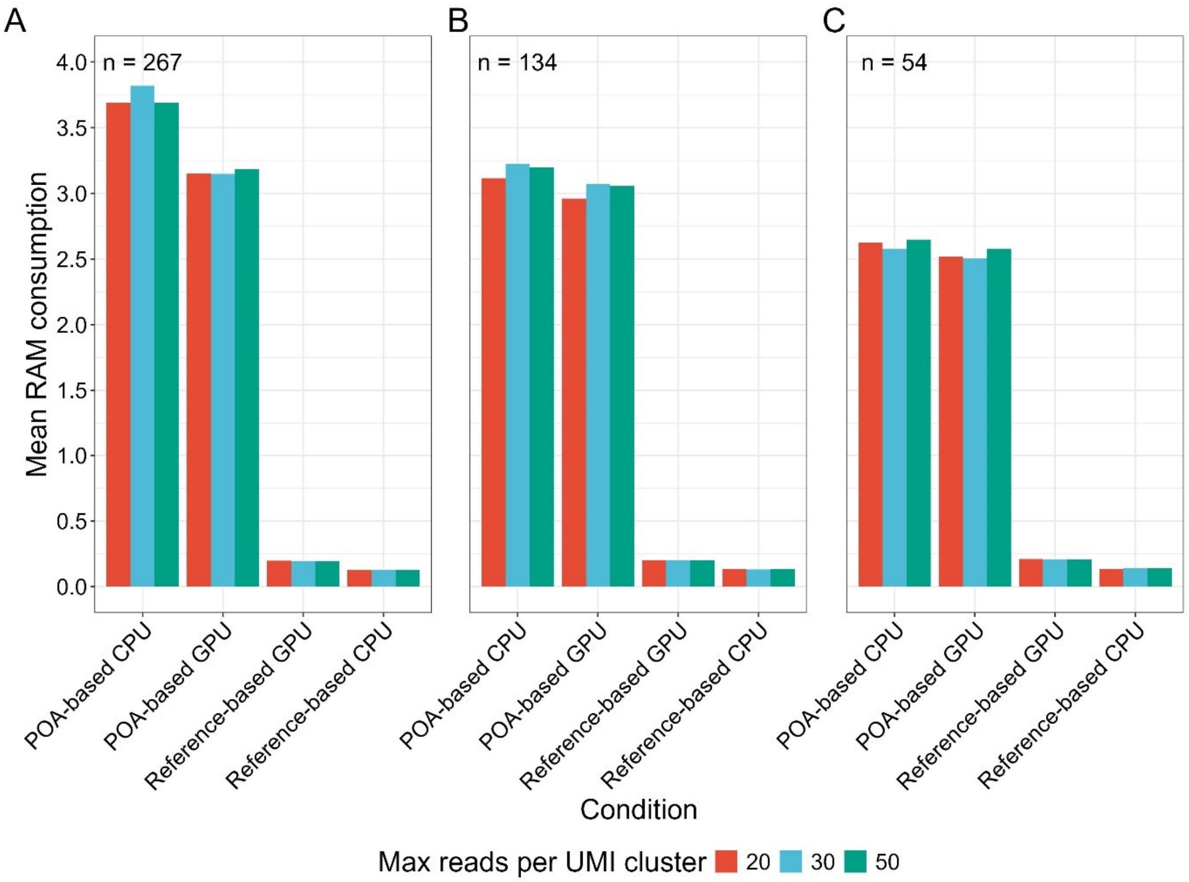

Supplementary figure 2. Mean RAM consumption of POA-based and reference-based polishing for GPU- and CPU mode (relative RAM consumption compared to CPU POA-based polishing are given in Supplementary table 2).

N is the number of polishing input files (i.e. the FASTA/FASTQ input files containing grouped UMI clusters for polishing). A-C) The mean RAM consumption for different configurations of the number of clusters per polishing input file (A: 10 clusters, B: 20 clusters, C: 50 clusters) and maximum number of reads per cluster (red bar [left]: 20, green bar [middle]: 30, blue bar [right]: 50). The maximum number of reads per cluster showed no systematic effect on RAM consumption. The number of clusters per polishing input file is inversely correlated with the RAM consumption for POA-based polishing modes, making larger file sizes more efficient. Reference-based polishing is 18 to 30-fold more memory efficient than the POA-based polishing.

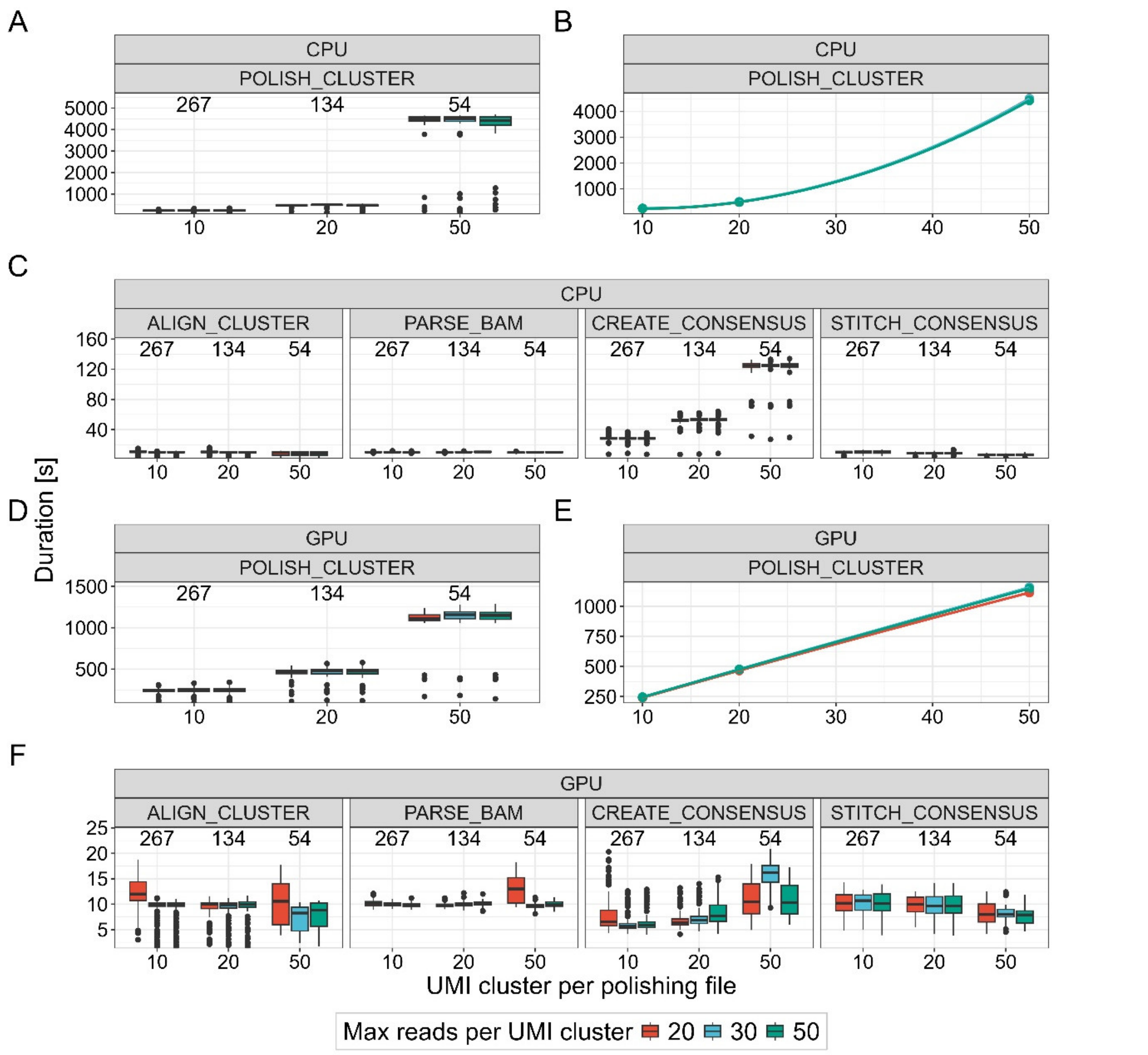

Supplementary figure 3. Execution time per polishing step of POA-based and reference-based polishing for CPU (A, B, C) and GPU mode (D, E, F) for different cluster numbers per polishing input file (10, 20 and 50 cluster per file) and different maximum read numbers per cluster (20, 30 and 50 reads per cluster). The number of processes evaluated is annotated above the boxplots.

For POA-based polishing in CPU mode (A, B), the median polishing time increased non-linearly with increasing number of clusters per polishing input file (250s, 500s and 4500s for 10, 20 and 50 cluster per file, respectively), while the number of reads per cluster shows no effect on polishing time. For the CPU reference-based polishing mode (B) times for alignment of the clusters to the reference (step: ALIGN_CLUSTER), parsing of the bam file (PARSE_BAM) and stitching of the consensus sequences (STITCH_CONSENSUS, i.e. merging polished chunks into a full-length consensus) are independent of file and cluster size. We observed a linear increase in process duration for the polishing step (CREATE_CONSENSUS; 25s, 50s and 125s for 10, 20 and 50 cluster per file). GPU acceleration of POA-based polishing (C and D) leads to a linear increase in polishing time, independent of the number of clusters per input file, requiring 250s, 500s and ≈1,100s for 10, 20 and 50 cluster per file. Finally, for GPU accelerated, reference-based polishing (D) all four polishing steps show a substantially reduced, near constant runtime independent of the file size (5-10s per CREATE_CONSENSUS step).

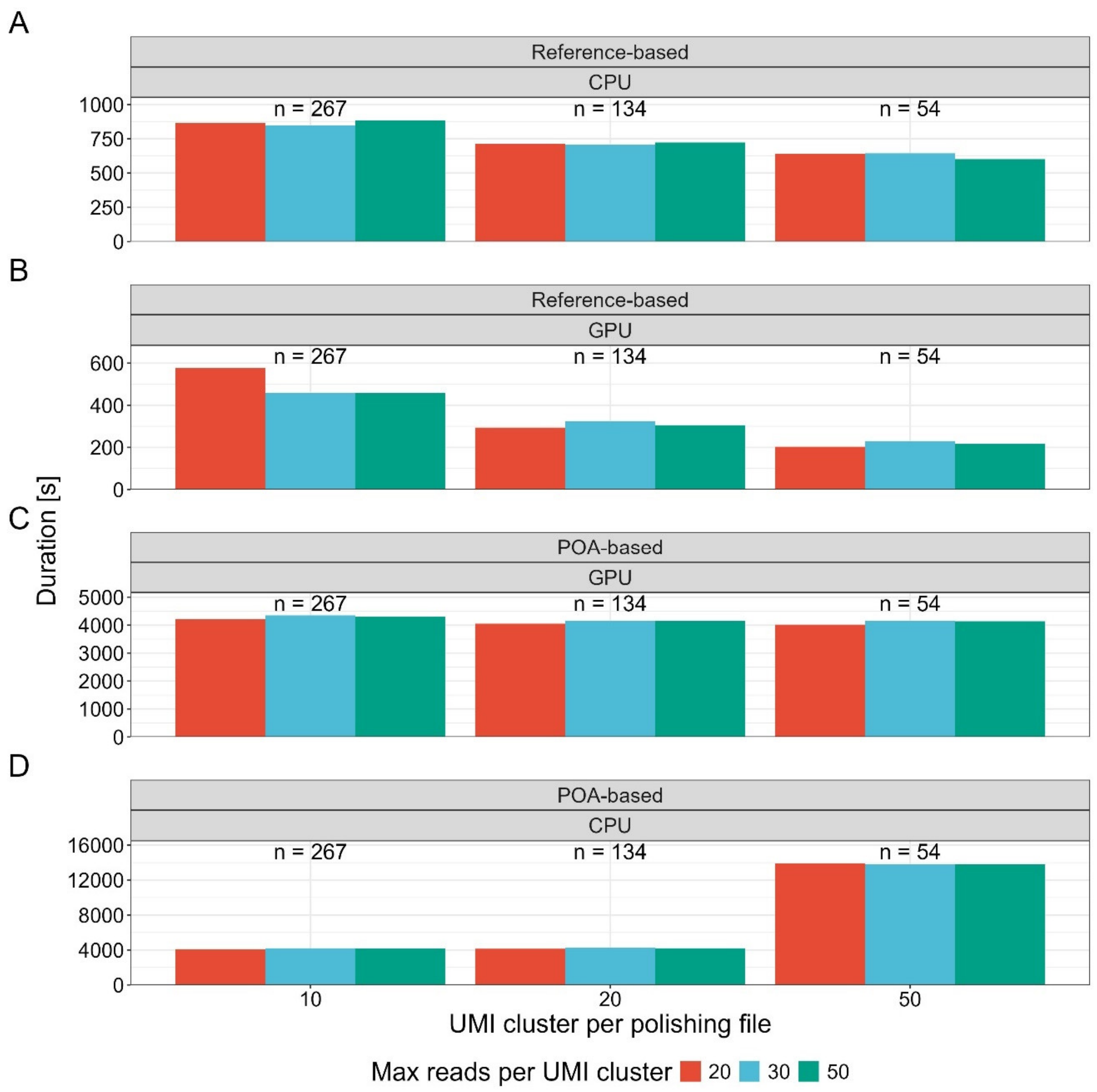

Supplementary figure 4. Total pipeline execution time (in seconds) for different conditions of reference-based and POA-based polishing in GPU-mode or CPU-mode.

Bars represent the total execution time grouped by the number of clusters per polishing input file (10, 20 and 50 cluster) and colored by the maximum number of reads per cluster (20 [red], 30 [blue] and 50 reads [turquoise]). N is the number of polishing input files. Reference-based polishing (A and B) was up to 20 times faster than POA-based polishing (C and D). For both CPU (A), and GPU (B) reference-based polishing modes increasing the number of UMI clusters per polishing input file led to a reduction of total execution time from 900s to 600s (CPU) and 600s to 200s (GPU). The total runtime of GPU, POA-based polishing remained constant for all conditions, taking about 4,000s. Only for CPU, POA-based polishing the execution time increased substantially up to 14,000s for 50 cluster per polishing input file.

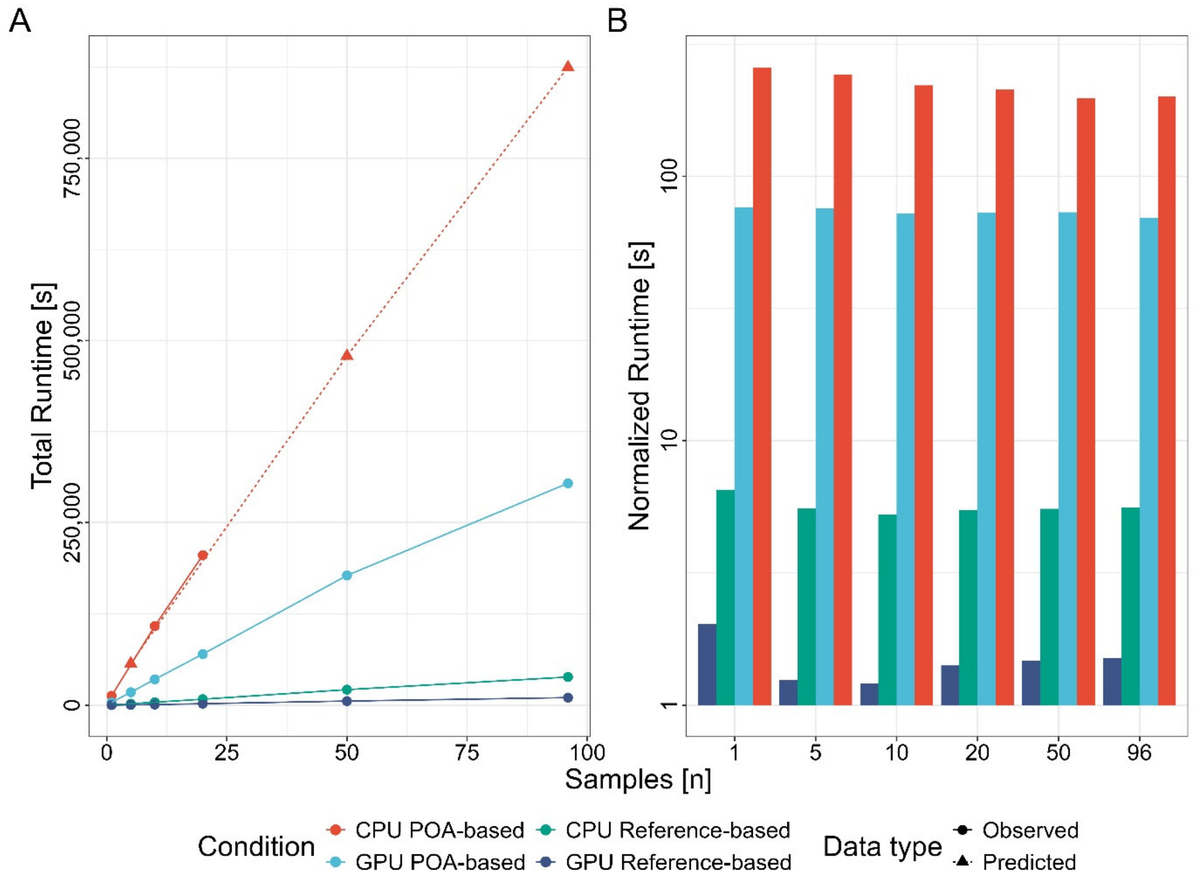

Supplementary figure 5. Total and normalized pipeline runtime for 1, 5, 10, 20, 50, and 96 samples using Reference- and POA-based polishing in GPU- or CPU-mode.

Due to its long execution times, CPU POA-based polishing was executed for 1, 10 and 20 samples (red dots) and execution time for 5, 50 and 96 samples was extrapolated using a log-log regression accounting for a potential non-linear scaling (red triangles with dashed line).

Panel A shows the total execution time of the pipeline. The total execution time increases linearly with the number of samples and clusters. Panel B shows the normalized runtime for the different analyses strategies. Normalizing the total runtime by the number of processes per sample shows that the execution time per sample remains roughly constant with increasing sample size. Reference-based polishing reduced the pipeline execution time by about 70-fold compared to POA-based polishing.

A

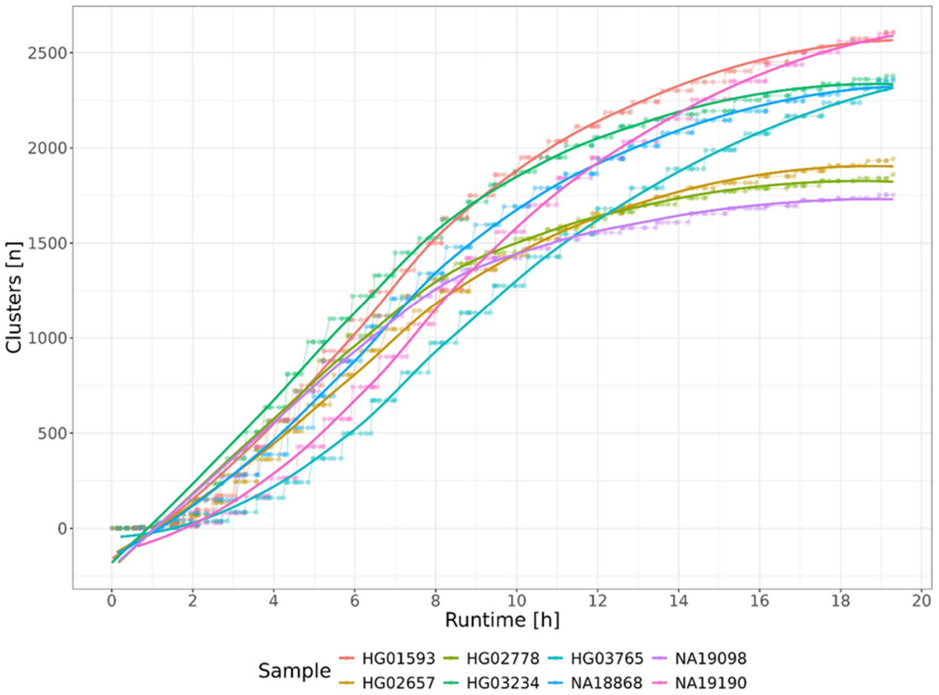

B

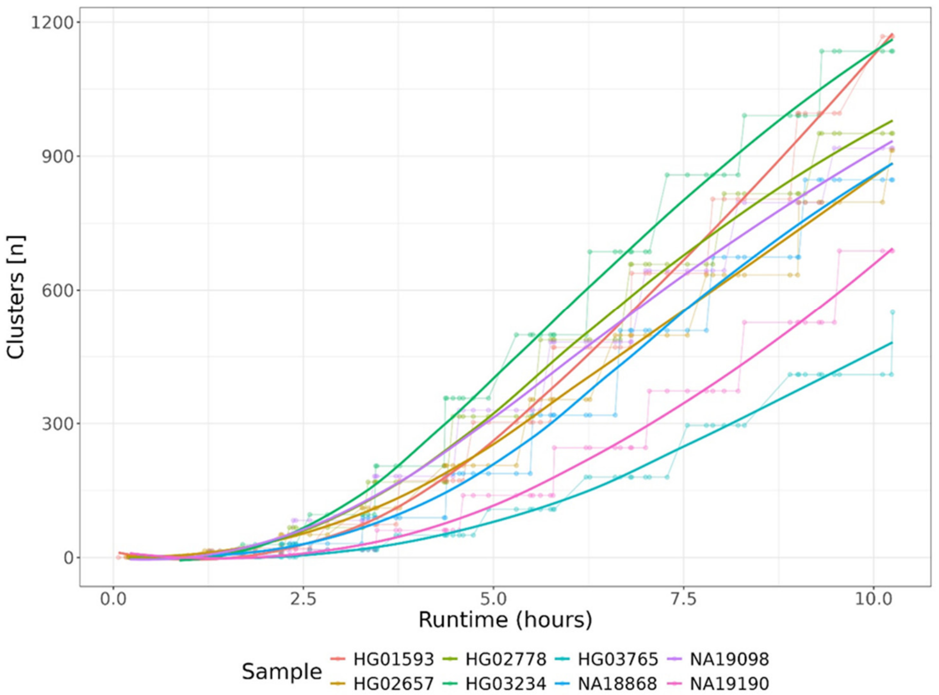

Supplementary figure 6. Cumulative number of clusters detected over sequencing runtime for each sample. Runtime (hours) is on the x-axis and the cumulative count of clusters (n) is on the y-axis. Points and semi-transparent lines show observed values at successive time points for individual samples (colored by sample), and the solid line per sample is a smoothed trend (LOESS; no confidence band). A: Full run. B: Eary stopping run using the real-time clustering function.
Please note that the multiple points within each “step” of a sample’s curve represent updates to the cluster file, which are triggered whenever a new FASTQ file is written for any sample. When a new FASTQ file is written for a specific sample, a new “step” in that sample’s curve is initiated.

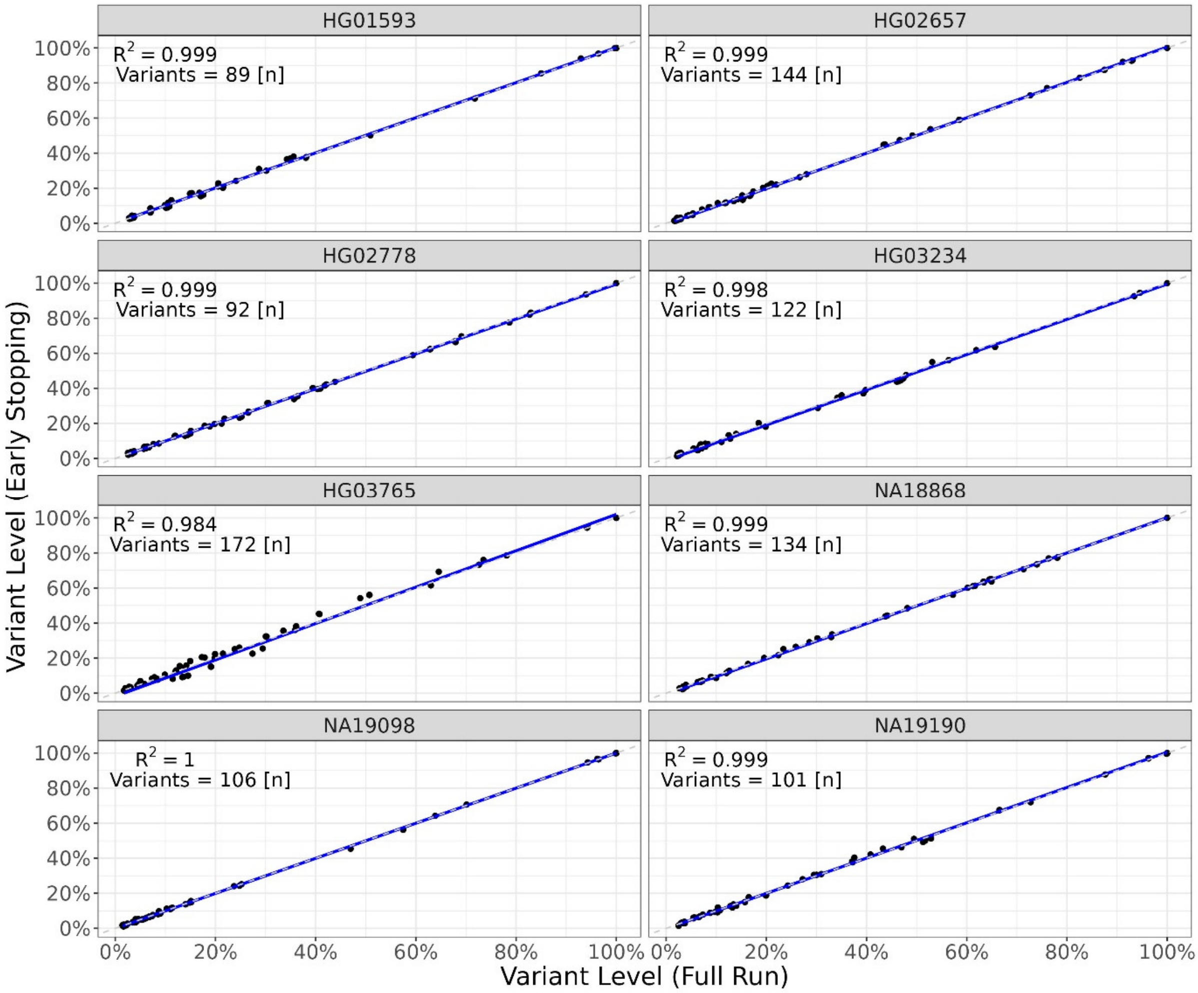

Supplementary figure 7. Variant-level concordance between early stopping and full run across eight samples. Each facet plots the variant-level frequency from the full run against the early-stopping run. Points represent individual variants; the solid blue line is the least-squares fit, and the grey dashed line indicates the perfect identity. The per-sample coefficient of determination (R^2) from a linear model and the number of variants are reported in each panel. Both runs resulted in the same number of variants and variant levels were strongly correlated (R^2 = 0.999±0.003; mean±SD).

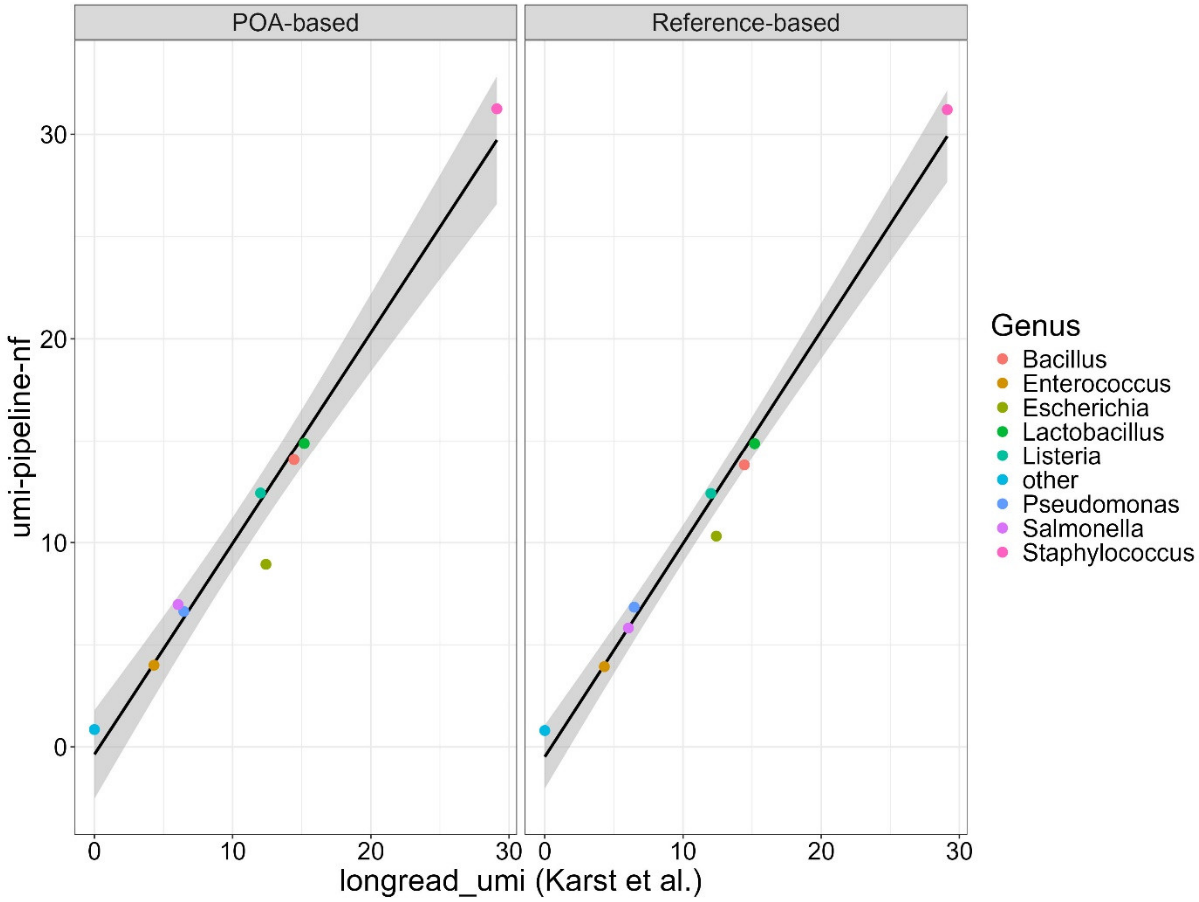

Supplementary figure 8. Correlation of umi-pipeline-nf and longread_umi for species abundance estimation of the mock microbial community from ZymoBIOMICS containing eight bacterial species for both polishing strategies of umi-pipeline-nf with GPU acceleration. Both polishing strategies of umi-pipeline-nf were highly correlated with the results obtained from the longread_umi. R^2=0.971 for POA-based polishing and R^2=0.985 for reference-based polishing.

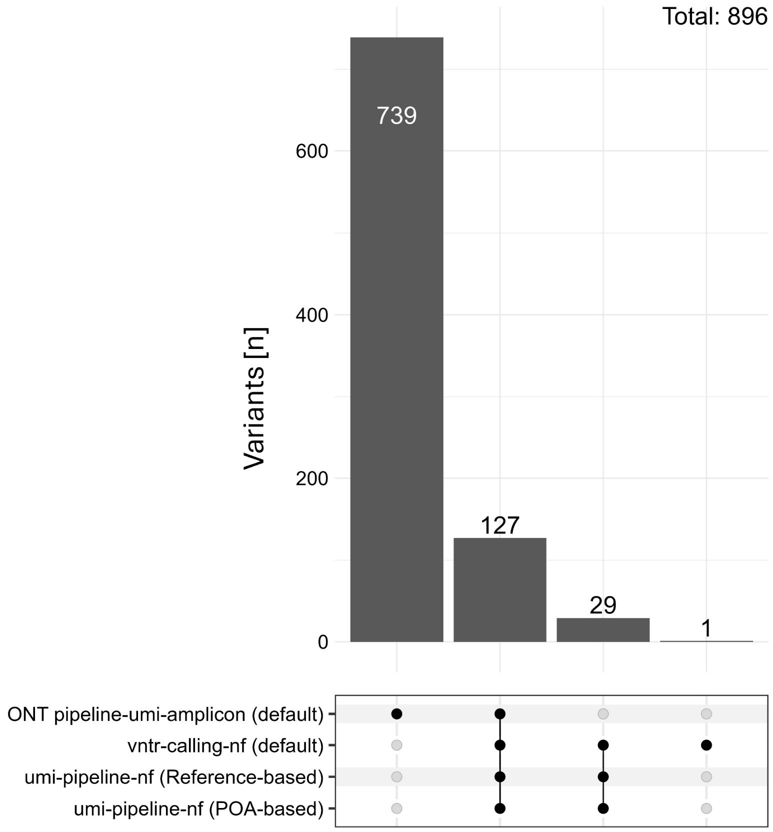

Supplementary figure 9. Overlap of variant detection between Oxford Nanopore Technologies (ONT) pipeline-umi-amplicon and our umi-pipeline-nf, as well as vntr-calling-nf[17] as non-UMI based reference method for low level variant calling in VNTR regions.

The dataset represents a 5.1 kb long amplicon mixture representing all KIV-2 repeat units of the *LPA* VNTR of an individual, as described in the main text and in [20]. Default settings were applied to each pipeline. Each bar represents the number of variants reported by the pipelines in each specific pipeline combination, highlighting both shared and unique variant calls between pipelines.

Of 896 variants, 127 variants were detected in all four settings. Both umi-pipeline-nf configurations and vntr-calling-nf shared another 29 variants, not reported by ONT's pipeline-umi-amplicon. In contrast, pipeline-umi-amplicon detected 739 additional variants at very low levels, which were not confirmed by others and most plausibly represent false-positives. One variant was reported solely by vntr-calling-nf.

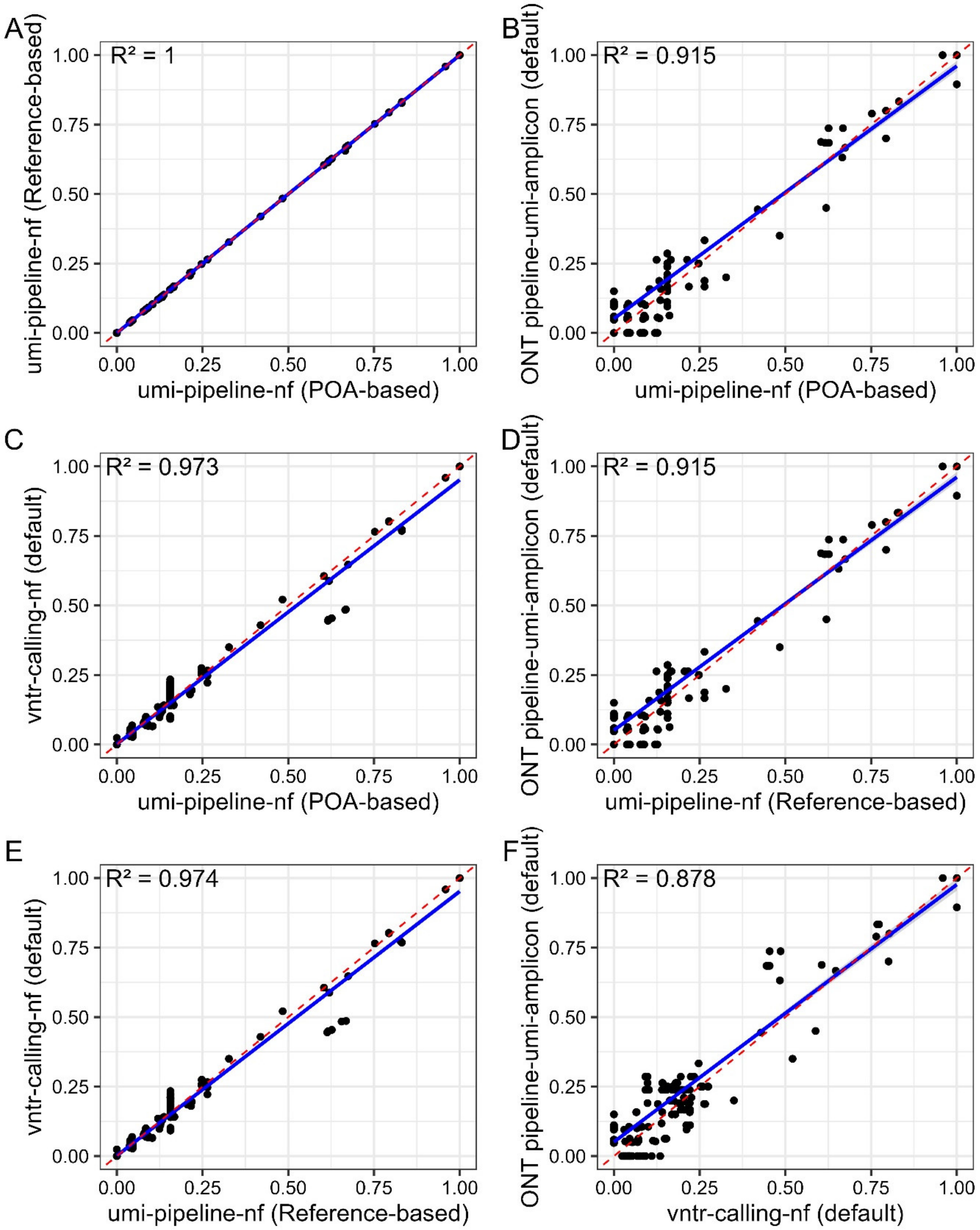

Supplementary figure 10. Correlation of variant level estimates between ONT's pipeline-umi-amplicon and our umi-pipeline-nf, as well as vntr-calling-nf as reference method for low level variant calling in VNTR regions. A-F compare pairwise results from umi-pipeline-nf with POA-based polishing, umi-pipeline-nf with reference-based polishing, pipeline-umi-amplicon (default settings), and vntr-calling-nf (default settings). Each panel shows the linear regression fit (blue line) and the bisecting line (red dashed). R^2 values are annotated within each plot. While we observed strong correlations between pipelines across all pairwise comparisons, low-level variant detection differs between pipeline-umi-amplicon and the other three pipelines (B, D, F).

Supplementary tables

Supplementary table 1. Mean durations and x-fold speed-up compared to POA-based polishing with CPU-only execution. *x-fold speed-up is relative to CPU POA-based runtime at same configuration.

Polishing	Mode	Clusters/File	Max Reads/Cluster	Mean Duration [s]	Mean duration CPU POA-based [s]	x-fold speed-up
POA-based	CPU	10	20	232.8	232.8	1.00
Ref.-based	CPU	10	20	58.5	232.8	3.98
POA-based	CPU	10	30	236.7	236.7	1.00
Ref.-based	CPU	10	30	57.6	236.7	4.11
POA-based	CPU	10	50	236.9	236.9	1.00
Ref.-based	CPU	10	50	57.2	236.9	4.14
POA-based	CPU	20	20	469.7	469.7	1.00
Ref.-based	CPU	20	20	80.3	469.7	5.85
POA-based	CPU	20	30	481.7	481.7	1.00
Ref.-based	CPU	20	30	79.9	481.7	6.03
POA-based	CPU	20	50	472.9	472.9	1.00
Ref.-based	CPU	20	50	80.8	472.9	5.85
POA-based	CPU	50	20	4,036.5	4,036.5	1.00
Ref.-based	CPU	50	20	143.1	4,036.5	28.21
POA-based	CPU	50	30	4,030.3	4,030.3	1.00
Ref.-based	CPU	50	30	142.9	4,030.3	28.21
POA-based	CPU	50	50	4,008.1	4,008.1	1.00
Ref.-based	CPU	50	50	143.5	4,008.1	27.93

Supplementary table 2. Absolute and relative RAM consumption per polishing step across conditions. *Relative RAM is normalized to CPU POA-based for the same configuration.

Polishing	Mode	Clusters/File	Max Reads/Cluster	Mean RAM [MB]	Relative RAM reduction*
POA-based	CPU	10	20	3.7	1.00
POA-based	GPU	10	20	3.2	1.17
Ref.-based	CPU	10	20	0.1	28.53
Ref.-based	GPU	10	20	0.2	18.54
POA-based	CPU	10	30	3.8	1.00
POA-based	GPU	10	30	3.1	1.21
Ref.-based	CPU	10	30	0.1	29.79
Ref.-based	GPU	10	30	0.2	19.62
POA-based	CPU	10	50	3.7	1.00
POA-based	GPU	10	50	3.2	1.16
Ref.-based	CPU	10	50	0.1	28.65
Ref.-based	GPU	10	50	0.2	18.81
POA-based	CPU	20	20	3.1	1.00
POA-based	GPU	20	20	3.0	1.05
Ref.-based	CPU	20	20	0.1	23.31
Ref.-based	GPU	20	20	0.2	15.37
POA-based	CPU	20	30	3.2	1.00
POA-based	GPU	20	30	3.1	1.05
Ref.-based	CPU	20	30	0.1	24.37
Ref.-based	GPU	20	30	0.2	15.91
POA-based	CPU	20	50	3.2	1.00
POA-based	GPU	20	50	3.1	1.05
Ref.-based	CPU	20	50	0.1	24.02
Ref.-based	GPU	20	50	0.2	15.79
POA-based	CPU	50	20	2.6	1.00
POA-based	GPU	50	20	2.5	1.04
Ref.-based	CPU	50	20	0.1	19.16
Ref.-based	GPU	50	20	0.2	12.49
POA-based	CPU	50	30	2.6	1.00
POA-based	GPU	50	30	2.5	1.03
Ref.-based	CPU	50	30	0.1	18.64
Ref.-based	GPU	50	30	0.2	12.45
POA-based	CPU	50	50	2.6	1.00
POA-based	GPU	50	50	2.6	1.03
Ref.-based	CPU	50	50	0.1	19.16
Ref.-based	GPU	50	50	0.2	12.86

Supplementary table 3. Mean durations and x-fold speed-up compared to CPU POA-based. *x-fold Flower is relative to CPU POA-based runtime at same configuration.

Polishing	Mode	Clusters/File	Max Reads/Cluster	Mean Duration [s]	CPU POA [s]	x-Fold Faster
POA-based	CPU	10	20	232.8	232.8	1.00
POA-based	GPU	10	20	239.6	232.8	0.97
Ref.-based	CPU	10	20	58.5	232.8	3.98
Ref.-based	GPU	10	20	40.4	232.8	5.77
POA-based	CPU	10	30	236.7	236.7	1.00
POA-based	GPU	10	30	244.1	236.7	0.97
Ref.-based	CPU	10	30	57.6	236.7	4.11
Ref.-based	GPU	10	30	35.8	236.7	6.62
POA-based	CPU	10	50	236.9	236.9	1.00
POA-based	GPU	10	50	243.9	236.9	0.97
Ref.-based	CPU	10	50	57.2	236.9	4.14
Ref.-based	GPU	10	50	35.7	236.9	6.65
POA-based	CPU	20	20	469.7	469.7	1.00
POA-based	GPU	20	20	452.7	469.7	1.04
Ref.-based	CPU	20	20	80.3	469.7	5.85
Ref.-based	GPU	20	20	35.4	469.7	13.28
POA-based	CPU	20	30	481.7	481.7	1.00
POA-based	GPU	20	30	462.1	481.7	1.04
Ref.-based	CPU	20	30	79.9	481.7	6.03
Ref.-based	GPU	20	30	36.0	481.7	13.39
POA-based	CPU	20	50	472.9	472.9	1.00
POA-based	GPU	20	50	462.2	472.9	1.02
Ref.-based	CPU	20	50	80.8	472.9	5.85
Ref.-based	GPU	20	50	37.3	472.9	12.68
POA-based	CPU	50	20	4,036.5	4,036.5	1.00
POA-based	GPU	50	20	1,045.5	4,036.5	3.86
Ref.-based	CPU	50	20	143.1	4,036.5	28.21
Ref.-based	GPU	50	20	42.1	4,036.5	95.86
POA-based	CPU	50	30	4,030.3	4,030.3	1.00
POA-based	GPU	50	30	1,073.4	4,030.3	3.75
Ref.-based	CPU	50	30	142.9	4,030.3	28.21
Ref.-based	GPU	50	30	41.2	4,030.3	97.92
POA-based	CPU	50	50	4,008.1	4,008.1	1.00
POA-based	GPU	50	50	1,066.3	4,008.1	3.76
Ref.-based	CPU	50	50	143.5	4,008.1	27.93
Ref.-based	GPU	50	50	36.5	4,008.1	109.75

Supplementary table 4. Mutserve variant level (i.e. fraction of *LPA* KIV-2 repeat units carrying a certain mutation) metrics across polishing strategies. SD = Standard deviation

Polishing	Mode	Clusters/File	Max Reads/Cluster	Number of variants [n]	Mean Variant Level [%]	SD Variant Level [%]
POA-based	CPU	10	20	156	19.778	20.991
POA-based	GPU	10	20	156	19.778	20.988
Ref.-based	CPU	10	20	156	19.783	20.962
Ref.-based	GPU	10	20	156	19.783	20.961
POA-based	CPU	10	30	156	19.776	20.988
POA-based	GPU	10	30	156	19.776	20.985
Ref.-based	CPU	10	30	156	19.784	20.963
Ref.-based	GPU	10	30	156	19.784	20.962
POA-based	CPU	10	50	156	19.776	20.988
POA-based	GPU	10	50	156	19.776	20.985
Ref.-based	CPU	10	50	156	19.784	20.963
Ref.-based	GPU	10	50	156	19.784	20.962
POA-based	CPU	20	20	156	19.778	20.991
POA-based	GPU	20	20	156	19.778	20.988
Ref.-based	CPU	20	20	156	19.783	20.962
Ref.-based	GPU	20	20	156	19.783	20.961
POA-based	CPU	20	30	156	19.776	20.988
POA-based	GPU	20	30	156	19.776	20.985
Ref.-based	CPU	20	30	156	19.784	20.963
Ref.-based	GPU	20	30	156	19.784	20.962
POA-based	CPU	20	50	156	19.776	20.988
POA-based	GPU	20	50	156	19.776	20.985
Ref.-based	CPU	20	50	156	19.784	20.963
Ref.-based	GPU	20	50	156	19.784	20.962
POA-based	CPU	50	20	156	19.778	20.991
POA-based	GPU	50	20	156	19.778	20.988
Ref.-based	CPU	50	20	156	19.783	20.962
Ref.-based	GPU	50	20	156	19.783	20.961
POA-based	CPU	50	30	156	19.776	20.988
POA-based	GPU	50	30	156	19.776	20.985
Ref.-based	CPU	50	30	156	19.784	20.963
Ref.-based	GPU	50	30	156	19.784	20.962
POA-based	CPU	50	50	156	19.776	20.988
POA-based	GPU	50	50	156	19.776	20.985
Ref.-based	CPU	50	50	156	19.784	20.963
Ref.-based	GPU	50	50	156	19.784	20.962

Supplementary table 5. Total and normalized pipeline runtime across increasing number of samples for different polishing strategies. The total runtime was normalized by the number of clusters per condition. For CPU POA-based polishing the pipeline was executed for 1, 10 and 20 samples and for 5, 50 and 96 samples execution time was extrapolated using a log-log regression accounting for a potential non-linear scaling.

Polishing	Mode	Samples [n]	Clusters [n]	Type	Total Runtime [s]	Normalized Runtime [s/task]
POA-based	CPU	1	50	Observed	12,920.54	258.41
POA-based	CPU	5	235	Predicted	57,116.82	243.05
POA-based	CPU	10	491	Observed	108,357.16	220.69
POA-based	CPU	20	961	Observed	205,421.84	213.76
POA-based	CPU	50	2,426	Predicted	478,856.60	197.39
POA-based	CPU	96	4,368	Predicted	874,616.31	200.23
POA-based	GPU	1	50	Observed	3,804.19	76.08
POA-based	GPU	5	235	Observed	17,808.36	75.78
POA-based	GPU	10	491	Observed	35,460.75	72.22
POA-based	GPU	20	961	Observed	70,050.28	72.89
POA-based	GPU	50	2,426	Observed	177,608.21	73.21
POA-based	GPU	96	4,368	Observed	303,799.47	69.55
Ref.-based	CPU	1	78	Observed	507.54	6.51
Ref.-based	CPU	5	378	Observed	2,088.08	5.52
Ref.-based	CPU	10	786	Observed	4,112.03	5.23
Ref.-based	CPU	20	1,524	Observed	8,292.65	5.44
Ref.-based	CPU	50	3,880	Observed	21,364.78	5.51
Ref.-based	CPU	96	6,954	Observed	38,624.64	5.55
Ref.-based	GPU	1	78	Observed	157.82	2.02
Ref.-based	GPU	5	378	Observed	469.25	1.24
Ref.-based	GPU	10	786	Observed	949.47	1.21
Ref.-based	GPU	20	1,524	Observed	2,152.78	1.41
Ref.-based	GPU	50	3,880	Observed	5,715.65	1.47
Ref.-based	GPU	96	6,954	Observed	10,445.28	1.50

Supplementary table 6. Cluster stats per sample and sequencing time of a full sequencing run compared to early stopping of the sequencing based on cluster numbers.

Sample	Runtime (Full Run, hours)	Clusters (Full Run)	Runtime (Early Stopping, hours)	Clusters (Early Stopping)	Relative Cluster number in Early Stopping (%)	Relative Runtime (%)
HG01593	19.3	2,607	10.25	1,168	45	53
HG02657	19.3	1,943	10.25	913	47	53
HG02778	19.3	1,861	10.25	951	51	53
HG03234	19.3	2,378	10.25	1,135	48	53
HG03765	19.3	2,354	10.25	550	23	53
NA18868	19.3	2,362	10.25	847	36	53
NA19098	19.3	1,754	10.25	918	52	53
NA19190	19.3	2,606	10.25	688	26	53

Supplementary table 7. Relative and total abundance of each bacteria found in the ZymoBIOMICS Microbial Community DNA Standard for both polishing strategies of the umi-pipeline-nf compared to the relative abundance calculated with longread_umi[21].

Genus	Relative Abundance Zymo [%]	Relative Abundance longread_umi [%]	Polishing umi-pipeline-nf	Relative Abundance umi-pipeline-nf [%]	Abundance umi-pipeline-nf [n reads]	Total Reads
Bacillus	17.4	14.44	POA-based	14.07	6,374	45,303
Bacillus	17.4	14.44	Ref.-based	13.81	6,568	47,547
Enterococcus	9.9	4.31	POA-based	4.00	1,810	45,303
Enterococcus	9.9	4.31	Ref.-based	3.93	1,869	47,547
Escherichia	10.1	12.41	POA-based	8.94	4,049	45,303
Escherichia	10.1	12.41	Ref.-based	10.32	4,906	47,547
Lactobacillus	18.4	15.18	POA-based	14.88	6,739	45,303
Lactobacillus	18.4	15.18	Ref.-based	14.87	7,069	47,547
Listeria	14.1	12.02	POA-based	12.43	5,629	45,303
Listeria	14.1	12.02	Ref.-based	12.41	5,899	47,547
Pseudomonas	4.2	6.47	POA-based	6.63	3,004	45,303
Pseudomonas	4.2	6.47	Ref.-based	6.84	3,250	47,547
Salmonella	10.4	6.05	POA-based	6.97	3,159	45,303
Salmonella	10.4	6.05	Ref.-based	5.82	2,766	47,547
Staphylococcus	15.5	29.12	POA-based	31.25	14,155	45,303
Staphylococcus	15.5	29.12	Ref.-based	31.21	14,841	47,547
other	0.0	0.00	POA-based	0.85	384	45,303
other	0.0	0.00	Ref.-based	0.80	379	47,547

Supplementary table 8 SARS-CoV-2 viral lineage assignment (Nextclade)[18].

Sample	Clade (Strain)	Consensus sequences per clade [n]	Consensus sequences per clade [%]	Total consensus sequences per sample [n]
patient1	21I (Delta)	61	8.69	702
patient1	21J (Delta)	641	91.31	702
patient2	21J (Delta)	828	100.00	828
patient3	21J (Delta)	937	100.00	937
patient4	21J (Delta)	436	100.00	436

Supplementary table 9 SARS-CoV-2 viral lineage assignment of patient1 including subclades (from Nextclade)[18].

Clade (Strain)	Carries A222V	Carries V367L	Carries G142D	Carries I834V	Carries I882	Consensus sequences per clade [n]	Consensus sequences per clade [%]	Total consensus sequences [n]
21I (Delta)	true	false	true	false	false	2	0.28	702
21I (Delta)	false	true	true	false	false	3	0.43	702
21I (Delta)	false	false	true	false	false	25	3.56	702
21J (Delta)	false	false	false	false	false	29	4.13	702
21I (Delta)	true	true	true	false	false	31	4.42	702
21J (Delta)	false	false	true	true	false	51	7.26	702
21J (Delta)	false	false	true	false	false	561	79.91	702

Supplementary references

1. Rescheneder P. catfishq. Accessed 2025-07-24, https://github.com/philres/catfishq

2. lh3. seqtk: Toolkit for processing sequences in FASTA/Q formats. Updated 2025–07–16. Accessed 2025-07-16, 2025. https://github.com/lh3/seqtk

3. Li H. Minimap2: pairwise alignment for nucleotide sequences. *Bioinformatics*. 2018;34(18):3094–3100. doi:10.1093/bioinformatics/bty191

4. Amstler S, Streiter G, Pfurtscheller C, et al. Nanopore sequencing with unique molecular identifiers enables accurate mutation analysis and haplotyping in the complex lipoprotein(a) KIV-2 VNTR. *Genome Med*. 2024;16(1):117. doi:10.1186/s13073-024-01391-8

5. Rognes T, Flouri T, Nichols B, Quince C, Mahe F. VSEARCH: a versatile open source tool for metagenomics. *PeerJ*. 2016;4:e2584. doi:10.7717/peerj.2584

6. Lee C, Grasso C, Sharlow MF. Multiple sequence alignment using partial order graphs. *Bioinformatics*. 2002;18(3):452–64. doi:10.1093/bioinformatics/18.3.452

7. Garrison E, Marth G. Haplotype-based variant detection from short-read sequencing. *ArXiv [Preprint]*. 2012:arXiv:1207.3907. doi:10.48550/arXiv.1207.3907

8. Wilm A, Aw PPK, Bertrand D, et al. LoFreq: A sequence-quality aware, ultra-sensitive variant caller for uncovering cell-population heterogeneity from high-throughput sequencing datasets. *Nucleic Acids Research*. 2012;40(22):11189–11201. doi:10.1093/nar/gks918

9. Weissensteiner H, Forer L, Schönherr S. Mutserve. Accessed 2024-02-02, https://github.com/seppinho/mutserve

10. Oxford Nanopore Technologies. Medaka: Sequence correction provided by ONT Research. Accessed 2025-08-29, 2025. https://github.com/nanoporetech/medaka

11. Karst SM, Ziels RM, Kirkegaard RH, et al. High-accuracy long-read amplicon sequences using unique molecular identifiers with Nanopore or PacBio sequencing. *Nat Methods*. 2021;18(2):165–169. doi:10.1038/s41592-020-01041-y

12. Oxford Nanopore Technologies. pipeline-umi-amplicon. Accessed 2023-06-06, https://github.com/nanoporetech/pipeline-umi-amplicon

13. Amstler S. umi-pipeline-nf. Accessed 2025-07-16, https://github.com/AmstlerStephan/umi-pipeline-nf

14. Oxford Nanopore Technologies. Ligation sequencing amplicons – custom PCR UMI (SQK-LSK109). Accessed 28-08-2025, https://nanoporetech.com/document/custom-pcr-umi

15. Auton A, Brooks LD, Durbin RM, et al. A global reference for human genetic variation. *Nature*. 2015;526(7571):68–74. doi:10.1038/nature15393

16. Shen W, Le S, Li Y, Hu F. SeqKit: A Cross-Platform and Ultrafast Toolkit for FASTA/Q File Manipulation. *PLoS One*. 2016;11(10):e0163962. doi:10.1371/journal.pone.0163962

17. Di Maio S, Zöscher P, Weissensteiner H, et al. Resolving intra-repeat variation in medically relevant VNTRs from short-read sequencing data using the cardiovascular risk gene LPA as a model. *Genome Biology*. 2024;25(1):167. doi:10.1186/s13059-024-03316-5

18. Hadfield J, Megill C, Bell SM, et al. Nextstrain: real-time tracking of pathogen evolution. *Bioinformatics*. 2018;34(23):4121–4123. doi:10.1093/bioinformatics/bty407

19. Zahm AM, Cranney CW, Gormick AN, et al. ConSeqUMI, an error-free nanopore sequencing pipeline to identify and extract individual nucleic acid molecules from heterogeneous samples. *bioRxiv*. 2025:2025.04.03.647077. doi:10.1101/2025.04.03.647077

20. Coassin S, Schönherr S, Weissensteiner H, et al. A comprehensive map of single-base polymorphisms in the hypervariable LPA kringle IV type 2 copy number variation region. *Journal of Lipid Research*. 2019;60(1):186–199. doi:10.1194/jlr.M090381

21. SorenKarst. longread_umi. Accessed 2025-07-16, https://github.com/SorenKarst/longread_umi
